# Conscious thought does not guide moment-to-moment actions—it serves social and cultural functions

**DOI:** 10.3389/fpsyg.2013.00478

**Published:** 2013-07-26

**Authors:** E. J. Masicampo, Roy F. Baumeister

**Affiliations:** ^1^Department of Psychology, Wake Forest UniversityWinston-Salem, NC, USA; ^2^Department of Psychology, Florida State UniversityTallahassee, FL, USA

**Keywords:** consciousness, conscious thought, action control, cultural evolution, unconscious

## Abstract

Humans enjoy a private, mental life that is richer and more vivid than that of any other animal. Yet as central as the conscious experience is to human life, numerous disciplines have long struggled to explain it. The present paper reviews the latest theories and evidence from psychology that addresses what conscious thought is and how it affects human behavior. We suggest that conscious thought adapts human behavior to life in complex society and culture. First, we review research challenging the common notion that conscious thought directly guides and controls action. Second, we present an alternative view—that conscious thought processes actions and events that are typically removed from the here and now, and that it indirectly shapes action to favor culturally adaptive responses. Third, we summarize recent empirical work on conscious thought, which generally supports this alternative view. We see conscious thought as the place where the unconscious mind assembles ideas so as to reach new conclusions about how best to behave, or what outcomes to pursue or avoid. Rather than directly controlling action, conscious thought provides the input from these kinds of mental simulations to the executive. Conscious thought offers insights about the past and future, socially shared information, and cultural rules. Without it, the complex forms of social and cultural coordination that define human life would not be possible.

Humans enjoy a private, mental life that is richer and more vivid than that of any other animal (e.g., Damasio, 1999; Edelman, 2004; Suddendorf, 2006). Yet as central as the conscious experience is to human life, numerous disciplines have long struggled to explain it (e.g., Blackmore, 2005). The present paper reviews the latest theories and evidence from psychology that addresses what conscious thought is and how it affects human behavior.

Our focus is on the type of conscious experience that is unique to humans. A common practice in discussions of consciousness is to distinguish between two levels (e.g., Damasio, 1999; Edelman, 2004). The first level, phenomenal awareness, is shared by humans with most other animals. It comprises the experience of sensations, feelings, or qualia. The second level, conscious thought, is largely unique to humans and includes self-awareness, inner reflections, and deliberations. This paper reviews the latest research on the link between this second level of consciousness and human action. Earlier and more extensive treatment of these issues is available in Baumeister and Masicampo (2010) and Baumeister et al. (2011).

We suggest that conscious thought adapts human behavior to life in complex society and culture. First, we review research challenging the common notion that conscious thought directly controls action. Second, we present an alternative view—that conscious thought processes actions and events that are typically removed from the here and now, and that it indirectly shapes action to favor culturally adaptive responses. Third, we summarize recent empirical work on conscious thought, which generally supports this alternative view.

## Questioning conscious thought as the controller of action

A commonly held view assumes that conscious thought is in charge of behavior (e.g., Wegner, 2002). However, several decades of psychology research have challenged this notion. The findings have shown that conscious thought has limited access to the mind's inner workings, while revealing that the unconscious is capable of initiating and guiding behavior without help from conscious thought.

### The limitations of conscious thought

If conscious thought were in charge of behavior, then people could presumably report and explain their actions accurately. To the contrary, Nisbett and Wilson (1977) showed repeatedly that people who were asked to explain their actions would overlook factors that had demonstrably large influences on their behavior. People even denied those influences when asked about them directly. Thus, when people introspect about their behaviors, they seem incapable of retrieving accurate accounts of what they did and why.

Gazzaniga (2000) has suggested that people explain their behaviors by fabricating stories. In his research, brain damaged patients who could not explain their behaviors accurately were nevertheless quick to provide plausible, though obviously false, explanations for their actions. More recent research has demonstrated a similar phenomenon in normally functioning adults (Johansson et al., 2005). This work employed sleight of hand to dupe participants into explaining decisions they did not make. Most participants failed to notice that these were not their decisions. Furthermore, they had no problem providing quick and elaborate explanations for why they made them, even though the explanations could not have possibly been true. The general pattern thus seems to be that people are unaware of their own behaviors. If the conscious self cannot recognize its own actions, it is unlikely that it controls them.

A further limitation of conscious thought is that it is too slow to initiate behavior. Libet (1985) observed people as they decided to initiate simple motor movements. His data revealed that conscious choices were too delayed to be the true source of behavior. Unconscious processes, on the other hand, were much earlier indicators of action (in milliseconds; for a recent conceptual replication of Libet's work, see Soon et al., 2008). Even for more complex decisions, such as how to vote in an upcoming election, conscious decisions appear days after the unconscious has made up its mind (Galdi et al., 2008). According to these findings, the conscious self receives its information too late in the chain of events to be the initiator of behavior.

### The dynamic unconscious

Other work has revealed that unconscious processes are capable of initiating and guiding action, including for complex behaviors once thought to require conscious control. Bargh and his colleagues (Bargh and Chartrand, 1999; Bargh and Morsella, 2008) have argued that most human behaviors are initiated automatically and unconsciously in response to environmental cues. Many social motives and goals have been shown to operate in this way. The mere exposure to words related to achievement can trigger a range of motivated behaviors aimed at attaining mastery over later tasks (Bargh et al., 2001). Crucially, participants are typically unaware of these environmental influences on their behavior. Thus, the initiation and subsequent regulation of behavior occurs despite the person having no conscious awareness of the process, including for complex sets of actions.

Thus, the emerging view in recent decades has been that conscious thought is not the all-powerful controller of behavior that many perceive it to be (Pocket, 2004; Dijksterhuis et al., 2005). The conscious self is often mistaken about what it does and why. Furthermore, the unconscious seems capable of guiding much of what people do. If conscious thought affects human action, it is not in the manner of controlling moment-to-moment actions. Its influence on behavior must lie elsewhere.

## Conscious thought serves social and cultural functions

The above empirical work has prompted a revised understanding of how conscious thought relates to action (e.g., Wegner, 2002; Pocket, 2004). Some have speculated that there is no role for conscious thought in determining behavior (e.g., Dijksterhuis et al., 2005). In our more positive view, nature would not have equipped humankind with such a complex capacity as conscious thought if it did not serve an adaptive function. We propose that conscious thought may have powerful indirect effects on behavior even if it does not directly control it. Furthermore, given the uniquely human nature of conscious thought, we suggest it likely serves uniquely human needs—particularly, social and cultural ones.

### Social coordination and communication

We propose that conscious thought enables coordination with the social and cultural environment (Baumeister and Masicampo, 2010). Our thinking follows other perspectives that emphasize social pressures as the driver of uniquely human mental capacities. These have argued that primate intelligence evolved for the purpose of adapting to social life (Byrne and Whiten, 1988; Dunbar, 1998), with humans further evolving the motivation to understand others' mental states and to communicate their own mental states with others (Tomasello, 1999; Tomasello et al., 2005). We propose that this pressure to communicate with others transformed thinking from an individual capacity to a social one.

James (1890) famously asserted that “thinking is for doing.” We suggest that much of conscious thinking might instead (or also) be for talking. Consistent with that view, conscious thought and speech seem to emerge in complementary ways both in phylogeny and in development (see Baumeister and Masicampo, 2010). The link between conscious thought and speech is also observable among adults, in whom the full processing of language requires conscious thought (Greenwald and Liu, 1985), and conscious thinking suffers if inner speech is suppressed (Emerson and Miyake, 2003).

We suggest that conscious thought and communication afford numerous advantages. People who share their thoughts within a group can correct one another's mistakes, and so talking enables drawing on others' wisdom. People who communicate can also reach agreements with one another, taking into account others' intentions, knowledge, and resources. Thus, talking also allows for coordination and collaborative planning.

### Why communication requires conscious thought

The proposition that conscious thoughts are largely for communicating does not by itself explain how conscious thoughts influence action or why they need be conscious in the first place. One answer to these questions was provided by Morsella (2005) to explain phenomenal awareness, and his answer applies to conscious thought as well. He argued that consciousness allows for communication across disparate parts of the mind, so that inner conflicts can be resolved. For organisms with few motivations, responses to sensory input can be supplied with relatively little information processing. For humans and most animals, however, motivations co-occur and conflict. In these situations, different parts of the mind offer diverging prescriptions for behavior. One part urges the body to flee while another calls on it to stay put. A major function of consciousness is to broadcast incoming sensory input to the disparate parts of the mind so that multiple needs may be negotiated and an optimal course of action taken. Phenomenal consciousness allows conflicts originating from the physical environment to be resolved. In humans, we propose that conscious thought enables conflicts originating from society and culture to be resolved as well.

A second answer to the questions about the utility of consciousness is that conscious thought makes possible certain kinds of information processing that the unconscious cannot perform. Specifically, we see conscious thought as the place where the unconscious creates meaningful sequences of events or ideas. Language is one important example. The unconscious can process only single words, but conscious thought can combine words into meaningful sentences (Baars, 2002). Furthermore, the amount of information that can be communicated in sentences is infinitely more than the amount that can be captured in single words. It is only through the integrative serial processing afforded by conscious thought that the mind can combine simple concepts to produce novel conclusions. Indeed, we argue that a key function of conscious thought is to enable the unconscious to derive new insights from the information it already has.

Many types of thinking are made possible by conscious thought, and each provides a means for the unconscious to reach new conclusions without acquiring additional outside information. These include logical reasoning, quantification, and causal understanding (e.g., DeWall et al., 2008). As with language, each of these thought processes involves combining simple ideas in accordance with shared rules. Furthermore, we propose that each produces novel conclusions that can be communicated to others or incorporated into one's own decisions and behaviors.

These categories of sequential thought may seem non-social, but we argue that each is a cultural process. Each type of thought employs rules communicated within culture. And each allows individuals to operate successfully within the culture, whether it is used to cooperate with others, justify one's actions (Haidt, 2007), or argue (Mercier and Sperber, 2011).

### Translating conscious thought into action

Conscious thought influences action via mental simulation (Baumeister and Masicampo, 2010). Much of conscious thinking involves simulating non-present events (Kane et al., 2007; Killingsworth and Gilbert, 2010), as when people relive past experiences, anticipate desired futures, consume fiction, or daydream. Thus, conscious thought focuses frequently on non-present information rather than on current actions. Furthermore, mental simulations incorporate both of the features of conscious thought discussed above. They comprise meaningful sequences of events, at times incorporating the types of thinking already mentioned (e.g., logical reasoning, quantification, causality). And they allow for inner crosstalk and conflict resolution (e.g., Morsella, 2005). A person can imagine the outcome of engaging in a certain behavior, and the various parts of the mind can access the simulation, objecting as problems arise. By mentally simulating positive and negative behaviors and outcomes, individuals can learn to perform or avoid them (e.g., Grouios, 1992).

We suggest the power of conscious thought is not in the direct control of action, as common views assume. Rather, its power lies in processing information from society and culture. It takes in information, and it combines it into meaningful mental simulations constructed according to cultural rules. These simulations can be used to determine optimal outcomes or to rehearse optimal ways of behaving. Thus, conscious thought allows individuals to translate information from culture into socially adaptive responses.

## The experimental evidence for effects of conscious thought on behavior

We recently reviewed the literature for evidence of conscious causation of behavior (Baumeister et al., 2011). Our review surveyed experiments in which conscious thoughts were manipulated by random assignment and effects on outward behavior were measured. By the logic of experimental design, such findings indicate that the conscious thought caused behavior. We identified many such phenomena, which had the following three themes.

### Integration of behavior across time

There are numerous influences of past and future reflections on behavior. People who reflect on and analyze the past can benefit. Some reflect on prior traumas to gain useful insights about them, thereby facilitating healthy recoveries (Pennebaker and Chung, 2007). Others analyze past actions to explore how they might have behaved differently, inviting lessons for achieving more desired outcomes later (Epstude and Roese, 2008). Alternatively, people who imagine or mentally relive the past can prolong prior mindsets rather than move beyond them (Lyubomirsky et al., 2006). Imagining the past preserves and even amplifies prior emotions and motivations, thereby affecting later behavior. For example, ruminating about a prior, anger-provoking event can amplify anger (Ray et al., 2008) and incite aggression (Bushman et al., 2005).

Thoughts of the future are also influential and have self-regulatory benefits (e.g., Schacter and Addis, 2007). Conscious thoughts facilitate goal attainment by allowing people to set plans for their goals (Gollwitzer, 1999) and energizing people toward desired, future outcomes (Taylor et al., 1998; Oettingen et al., 2001, 2009). Thoughts of the future can also sway behavior by exposing people to the potential consequences of their actions. For example, anticipation of regret can sway decisions (Tetlock and Boettger, 1994; Zeelenberg et al., 1996; Zeelenberg and Beattie, 1997).

### Consideration of social and cultural factors

Conscious thought also enables people to connect with others. It enables perspective taking, which enhances social coordination and negotiation outcomes (Galinsky et al., 2008a,b). It also allows people to communicate effectively with others (Roßnagel, 2000), which promotes cooperation in groups (e.g., Dawes et al., 1977; Jorgenson and Papciak, 1981).

Conscious thought likewise allows people to modify their behaviors to adhere to group expectations, norms, and laws, usually to the benefit of both the individual and the group. When people think about and explain their actions, group decisions (Scholten et al., 2007) and joint negotiation outcomes improve (De Dreu et al., 2000), and interaction partners become more cooperative, less hostile, and more trusting (De Dreu et al., 2006). Even absent any specific interaction partners, conscious thought generally promotes doing what is morally right (Caruso and Gino, 2011; Amit and Greene, 2012).

### Selection from among multiple alternative options

We propose that conscious thought is particularly useful for allowing people to consider multiple possible actions or outcomes. This is evident in counterfactual thinking. People often cannot help but reflect on how they might have behaved differently in the past. Such thinking can inspire new, improved strategies for later behavior (Epstude and Roese, 2008).

Consideration of alternative actions is also apparent in self-regulation and decision making. Hofmann et al. (2009) noted that explicit preferences and automatic impulses are often in conflict, and that explicit preferences are likely to guide behavior when people are free to reflect. In contrast, when conscious reflection is hindered, people are more impulsive (Ward and Mann, 2000) and more likely to yield to external influences (Westling et al., 2006). Conscious thought thus promotes adopting non-automatic forms of responding.

Pursuit of alternative responses is evident as well in sports. In almost every popular sport, researchers have found that the mental rehearsal of motor skills is nearly as beneficial for performance as physical practice (Druckman and Swets, 1988; Driskell et al., 1994). Thus, conscious mental practice improves skilled performance.

Each of the above patterns suggests that conscious thought does indeed help cause behavior. In each case, the influence of conscious thought on action is mostly indirect. Conscious thought is generally not found to guide moment-to-moment actions. However, reflections on the past enable people to improve later behaviors, considerations of social or cultural information sway decisions in favor of more cooperative responses, and mental simulations of plans and skills can be used to reshape habits. These findings support the notion that conscious thought is slightly removed from present actions, but that it nevertheless provides influential input into behavior.

## Conclusion

The past several decades of research in psychology have revealed some important limitations of conscious thought. Specifically, the findings suggest that conscious thought is not the direct controller of behavior that many assume it to be. We have argued nonetheless that it plays a crucial role in shaping human behavior. Our approach assumes that uniquely human capacities evolved to solve uniquely human challenges (e.g., Baumeister, 2005). Other animals interact with the physical environment (i.e., action control) without needing the capacity for conscious thought (Roberts, 2002). Humans, however, face the unique challenge of striving in society and culture (Baumeister, 2005). We think that it is precisely for that purpose that conscious thought developed.

In our review of the empirical research on conscious thought, we found numerous kinds of evidence in support of this view (Baumeister et al., 2011). The findings suggest that conscious thought affects behavior indirectly, by integrating information across time and from culture, so that multiple alternative behaviors—particularly socially adaptive ones—can be considered and an optimal action selected.

We conclude that most or all of human behavior is likely a product of conscious and unconscious processes working together. The private daydreams, fantasies, and counterfactual thoughts that pervade everyday life are far from being a feckless epiphenomenon. We see these processes as the place where the unconscious mind assembles ideas so as to reach new conclusions about how best to behave, or what outcomes to pursue or avoid. Rather than directly controlling action, conscious thought provides the input from these kinds of mental simulations to the executive. Conscious thought offers insights about the past and future, socially shared information, and cultural rules. Without it, the complex forms of social and cultural coordination that define human life would not be possible.

### Conflict of interest statement

The authors declare that the research was conducted in the absence of any commercial or financial relationships that could be construed as a potential conflict of interest.
